# Parasites specific to centipedes form a new major lineage of terrestrial gregarines

**DOI:** 10.1038/s41598-024-83990-4

**Published:** 2025-01-02

**Authors:** Tatiana S. Miroliubova, Kirill V. Mikhailov, Timur G. Simdyanov, Vladimir V. Aleoshin, Đinh Thế Dũng, Aleksandra I. Kudriavkina

**Affiliations:** 1 Severtsov Institute of Ecology and Evolution, Russian Academy of Sciences, Leninsky Ave. 33, Moscow, Russian Federation 119071; 2 Joint Vietnam-Russia Tropical Science and Technology Research Center, Hanoi, Vietnam; 3 Belozersky Institute for Physico-Chemical Biology, Lomonosov Moscow State University, Leninskiye Gory 1, bldg. 40, Moscow, Russian Federation 119991; 4 Kharkevich Institute for Information Transmission Problems, Russian Academy of Sciences, Bolshoy Karetny Ln. 19, bldg. 1, Moscow, Russian Federation 127051; 5 Faculty of Biology, Lomonosov Moscow State University, Leninskiye Gory 1, bldg. 12, Moscow, Russian Federation 119234

**Keywords:** Phylogenetics, Taxonomy

## Abstract

Gregarines from the families Dactylophoridae and Trichorhynchidae parasitize exclusively centipedes and have a distinct morphology among other terrestrial eugregarines, but their evolutionary relationships have not yet been studied with molecular methods. Here we obtain rDNA operon sequences for the dactylophorids and trichorhynchids. We describe a new species *Trichorhynchus efeykini* sp. n. from a scutigeromorph *Thereuopoda longicornis* from Vietnam. Phylogenetic analyses with combined SSU, 5.8S and LSU rDNA dataset support the previously proposed separation of *Trichorhynchus* to the Trichorhynchidae based on morphology and recover the dactylophorids and trichorhynchids as sister groups in a monophyletic clade. This clade appears sister to the clade of the Actinocephaloidea and Stylocephaloidea, and represents a new major lineage of terrestrial gregarines that we designate as a new superfamily Dactylophoroidea.

## Introduction

Terrestrial gregarines are parasites that have been known for almost 200 years^1^. They infect a wide range of terrestrial and freshwater invertebrates, including annelids and arthropods ^2^. Metagenomic data shows their high abundance in soil communities, which points to their significant yet currently underestimated role in terrestrial ecosystems^3,4^. However, their evolutionary relationships remain largely unclear and taxonomy is still mainly based on morphological data, since many genera and even families do not have a single sequenced representative. Two of such families are Dactylophoridae Léger, 1892 and Trichorhynchidae Ormières, Marquès & Puisségur, 1977. Recent phylogenies have outlined four lineages of terrestrial gregarines, which have been designated as superfamilies Actinocephaloidea Léger, 1892, Gregarinoidea Labbé, 1899, Stenophoroidea Clopton, 2009, and Stylocephaloidea Clopton, 2009^5–8^, but it is unclear how dactylophorids and trichorhynchids are related to these superfamilies.

The members of Dactylophoridae and Trichorhynchidae parasitize exclusively centipedes (Chilopoda). The dactylophorids infect Geophilomorpha, Lithobiomorpha, and Scolopendromorpha centipedes, while the only described trichorhynchid, *Trichorhynchus pulcher* Schneider, 1882, infects Scutigeromorpha centipedes^2^.

Dactylophorid trophozoites have a remarkable distinctive feature—an asymmetrical and dilated protomerite carrying an attachment apparatus in the form of a number of digitiform or filiform processes—rhizoids, instead of a single epimerite as in the majority of eugregarines^9–11^. At first, *Trichorhynchus pulcher* was placed in the family Dactylophoridae as a centipede parasite^12^. Later, Ormières and colleagues studied the ultrastructure of *T*. *pulcher* trophozoites^13,14^ and found significant morphological differences from other dactylophorids, including the absence of rhizoids, a lobed epimerite anchored close to the basal lamina of the host intestinal epithelium, a well-developed neck—an elongated anterior end of protomerite deeply immersed in the host intestinal epithelium, and the presence of multiple cytopilia on the cell surface instead of a typical eugregarine epicyte (multiple pellicular folds)^2^. Based on this, they separated *Trichorhynchus* into a monotypic family Trichorhynchidae^13^. Yet in addition to the closely related hosts, there is another feature bringing Trichorhynchidae closer to Dactylophoridae: they have similar (but not identical) mechanisms of the gametocyst dehiscence involving a dilating cytoplasmic residuum, the pseudocyst^13,15^. In order to determine the relationship between the Dactylophoridae and Trichorhynchidae and their position among other gregarines, we obtained rDNA operon sequences of the dactylophorids *Echinomera hispida* (Schneider, 1876) Labbé, 1899 and *Grebnickiella gracilis* (Grebnicki, 1873) Bhatia, 1938 and a trichorhynchid *Trichorhynchus efeykini* sp. n. described here, and reconstructed a phylogeny of terrestrial gregarines.

## Results

**Fig. 1:**
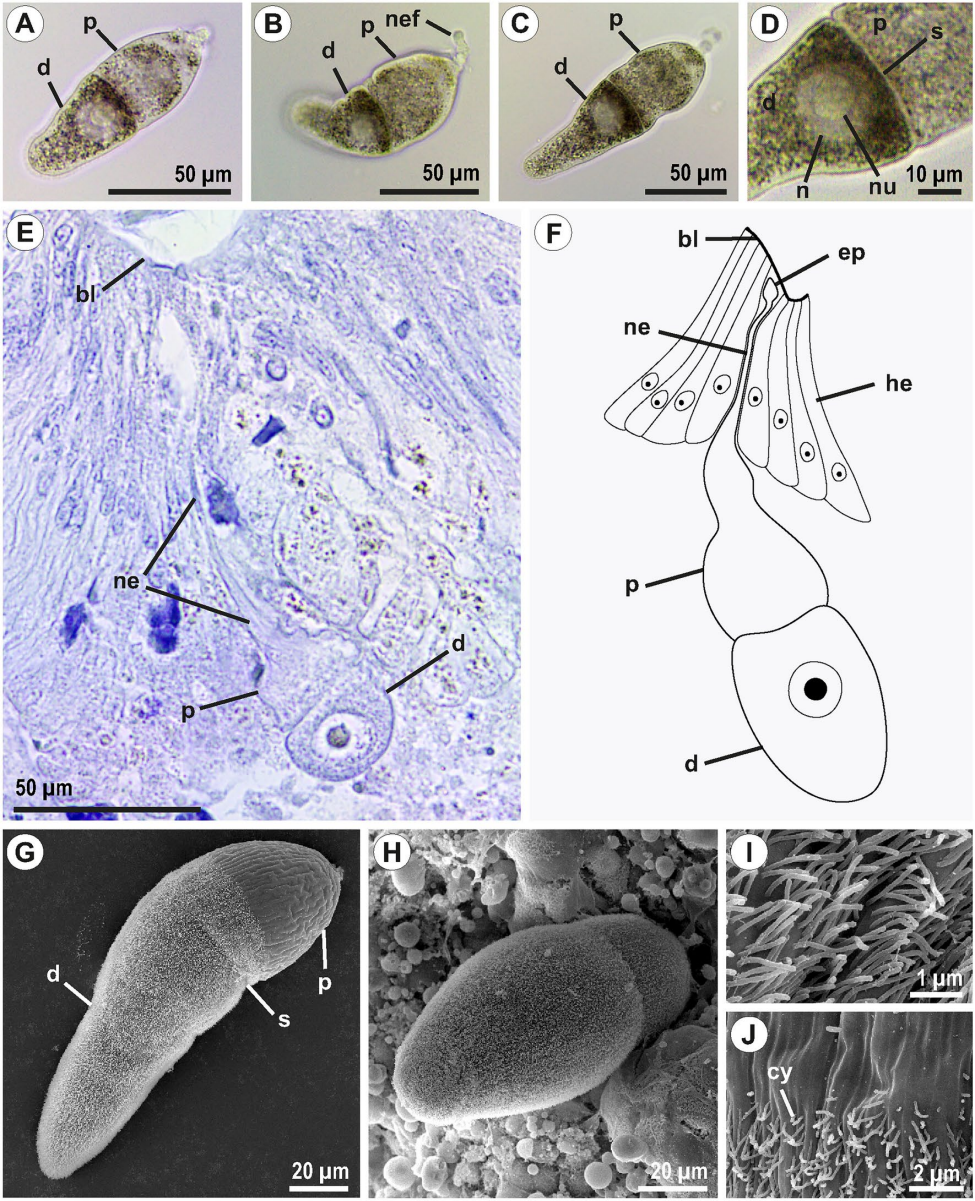
Morphology of *Trichorhynchus efeykini* sp. n. (**A**, **B**) young trophozoite; (**C**) trophozoite; (**D**) middle part of a trophozoite cell; (**E**) histological section of *Thereuopoda longicornis* intestinal epithelium with a *T. efeykini* trophozoite attached; (**F**) schematic drawing of an attached trophozoite reconstructed from a series of histological sections (see Supplementary fig. S1); (**G**) trophozoite, SEM; (**H**) attached trophozoite; (**I**) cell surface with numerous cytopilia; (**J**) the area of protomerite surface where the cytopilic carpet ends. bl—basal lamina; cy—cytopilia; d—deutomerite; ep—epimerite; he—host entherocyte; n—nucleus; nu—nucleolus; ne—neck; nef—neck fragment; p—protomerite; s—septum;

Hundreds of trophozoites of *Trichorhynchus efeykini* sp. n. were found in the intestine of one individual of *Thereuopoda longicornis* Fabricius, 1793. Narrowly obpyriform gregarines had a relatively large protomerite (the protomerite length to total length ratio was 1:2.5 to 1:3) and a deutomerite of the same width as the protomerite near the septum (Fig. 1A–G). There was a slight constriction along the septum (Fig. 1D,G). Trophozoites had an attachment apparatus in the form of a lobed epimerite located at the anterior end of an elongated neck and anchored close to the basal lamina of the host intestinal epithelium (Fig. 1B,E,F, and Supplementary fig. S1). Parasites had a spherical nucleus with a large eccentrically placed nucleolus, with the nucleolus to nucleus diameter ratio of 1:2 (Fig. 1D). The nucleus had variable position among individuals, and could be observed in any part of the deutomerite. The cell surface was covered with multiple cytopilia that were absent at the front part of protomerite adjacent to the host intestinal epithelium (Fig. 1G–J). Detached gregarines demonstrated gliding and sometimes bending motility. Detailed measurements of gregarines are given in Table 1.

**Table 1:** Measurements of *Trichorhynchus efeykini* (in μm).

*Trichorhynchus efeykini*, trophozoites							
n = 24	TL	PL	DL	PWM	PWE	DWM	DWE
Mean	149.7	53.9	95.8	50.4	48.4	48.8	39.85
Standard deviation	44.6	14.6	34.3	10.9	10.7	11.8	12.4
Standard error of the mean	9.1	3.0	7.0	2.2	2.2	2.4	2.5
Max	242.9	90.1	157	69.4	68.8	69	61.8
Min	85	37.5	46.8	32.4	29	29.5	23.2

TL—total length; PL—length of protomerite; DL—length of deutomerite; PWM—maximum width of protomerite; PWE—width of protomerite at equatorial axis; DWM—maximum width of deutomerite; DWE—width of deutomerite at equatorial axis; n—number of individuals.

**Fig. 2:**
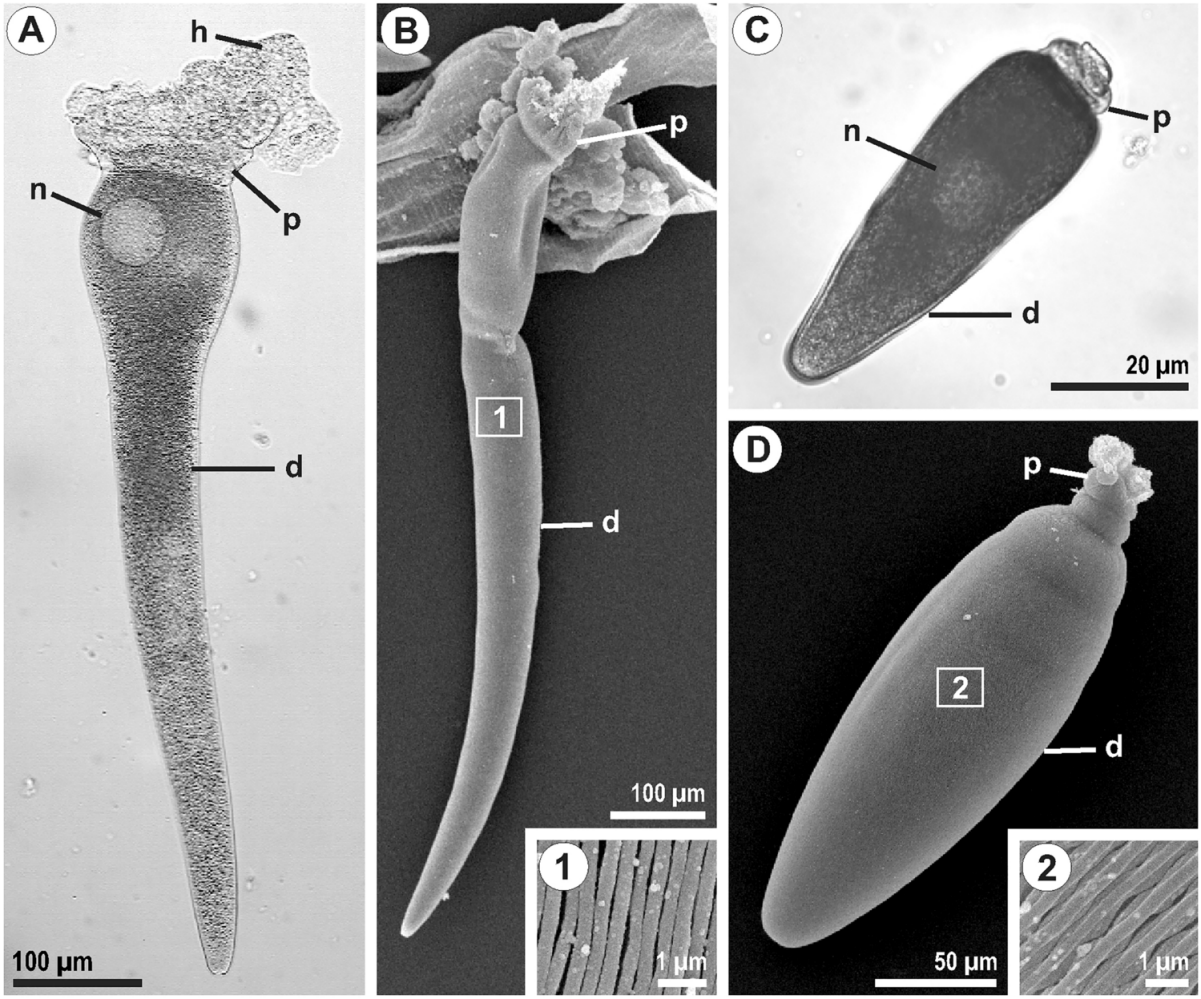
Morphology of *Grebnickiella gracilis* and *Echinomera hispida*. (**A**, **B**) trophozoite of *G*. *gracilis*; inset 1—epicytic folds of *G*. *gracilis*; (**C**, **D**) trophozoite of *E*. *hispida*, isolate TP; inset 2—epicytic folds of *E*. *hispida*. d—deutomerite; h—host gut epithelium residuum attached to rhizoids; n—nucleus; p—protomerite;

Three out of four collected individuals of *Scolopendra cingulata* were infected with 3–100 trophozoites of *Grebnickiella gracilis*. Nine out of nineteen individuals of *Lithobius forficatus* from Timiryazevsky Park were infected with 1–50 trophozoites of *Echinomera hispida* (TP isolate). Two out of five individuals of *L*. *forficatus* from the Zvenigorod Biological Station were infected with 1–7 trophozoites of *E*. *hispida* (ZBS isolate). The TP and ZBS isolates of *E*. *hispida* had an identical morphology. The trophozoites of *Grebnickiella gracilis* and *Echinomera hispida* had an appearance typical for the species, with asymmetrical protomerite and epicytic folds on the cell surface (Fig. 2).

Near-complete sequences of the rRNA operon (SSU rDNA, ITS1, 5.8S rDNA, ITS2, and LSU rDNA) were obtained for *Echinomera hispida* ZBS isolate (5522 bp), *Grebnickiella gracilis* (5145 bp), and *Trichorhynchus efeykini* sp. n. (5563 bp); GenBank accessions: PP657469, PP654190, PP657470. Partial sequence of SSU rDNA (1595 bp) was obtained for *Echinomera hispida* TP isolate; GenBank accession: PP659854.

**Fig. 3:**
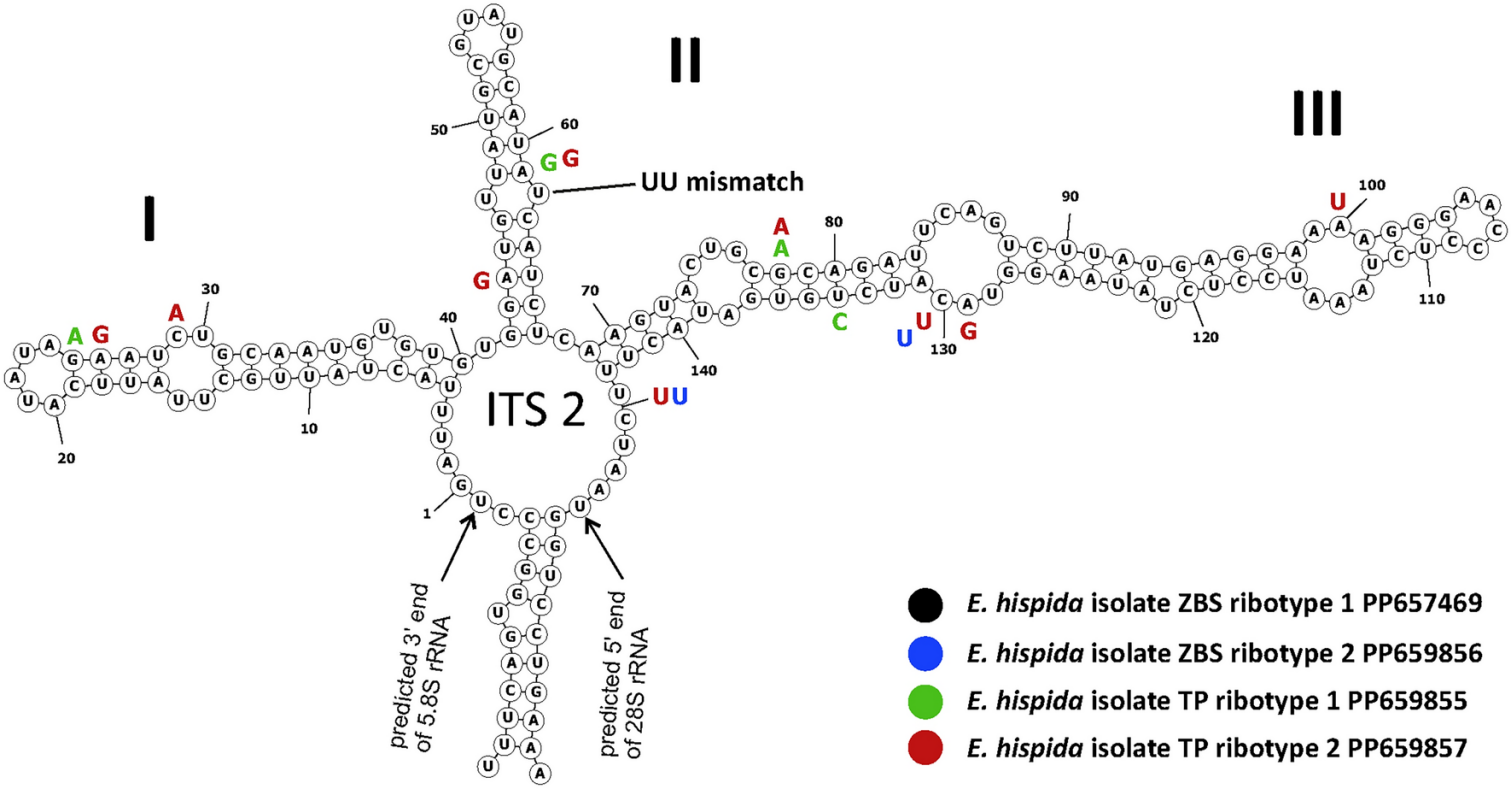
Predicted secondary structure of the ITS2 transcripts of *Echinomera hispida* demonstrating differences between the four ribotypes (in color). Helices are numbered I–III.

*Echinomera hispida* TP and ZBS isolates differed slightly in the SSU rDNA sequences (the identity percentage was 99.62%). In order to determine the degree of their relationship, we obtained their ITS2 sequences and searched for compensatory base changes in the secondary structures of the ITS2. The presence of compensatory base changes in the secondary structures of ITS2 regions is a criterion for separating cryptic species^16–19^. We identified a total of four ribotypes in *E. hispida*: two in the TP isolate and two in the ZBS isolate (Fig. 3); GenBank accessions: PP659855, PP659857, PP657469, PP659856. The secondary structure of the ITS2 region of *E. hispida* isolates consists of three helices, similarly to some other sporozoans^8,20^, and the second helix has a typical U-U mismatch^21^. Polymorphism in the ZBS isolates could only be searched from nucleotide position 120 to the end of the ITS2 due to low coverage in the transcriptomic data. The handful of differences found in the secondary structure of the ITS2 regions did not represent compensatory base changes, indicating both TP and ZBS isolates belong to the same species.

**Fig. 4:**
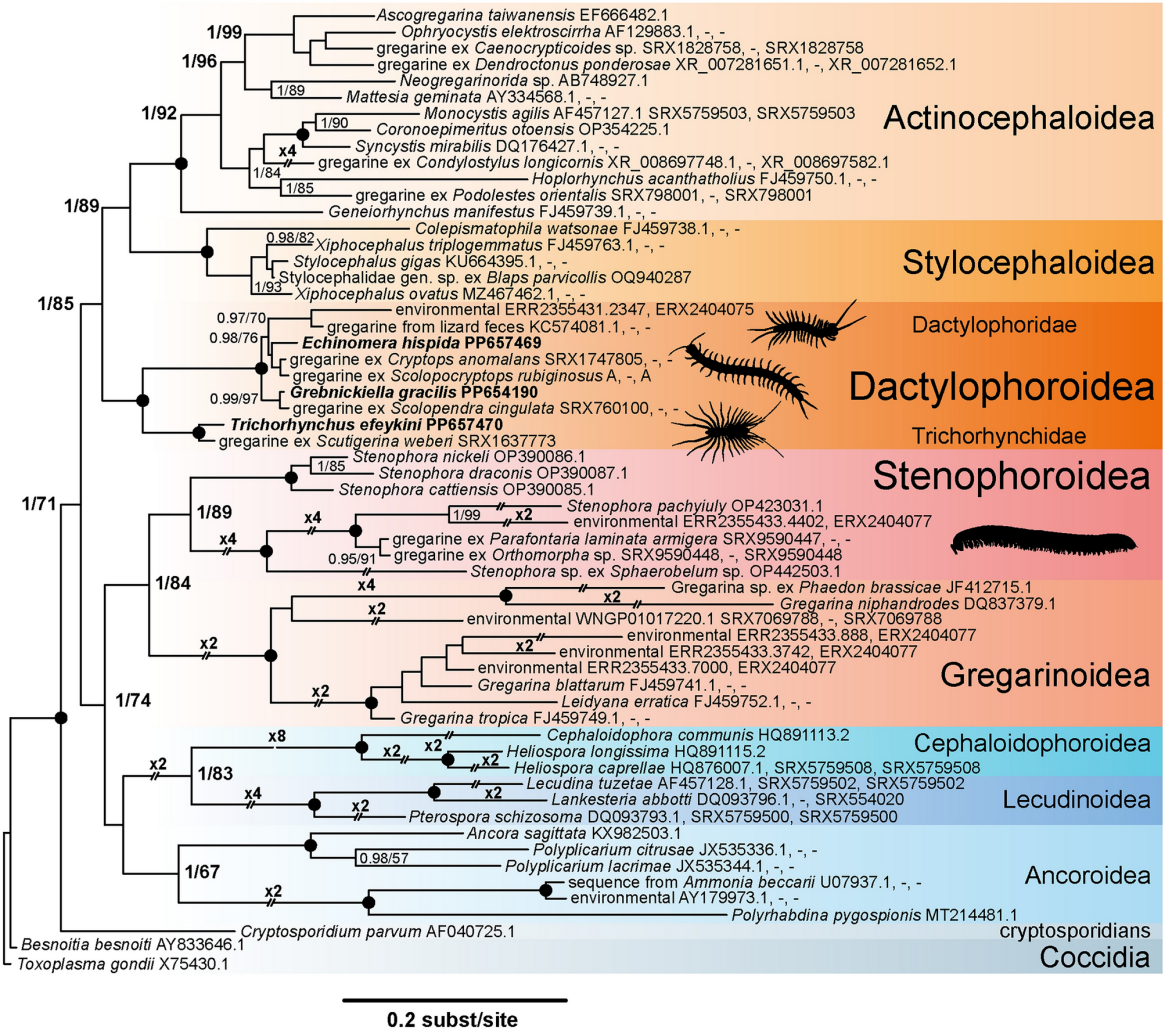
Bayesian inference tree of eugregarines with an alignment of 59 concatenated SSU, 5.8S and LSU rDNA sequences (4329 sites). Tree node support values: Bayesian posterior probabilities (PP, left) and ML bootstrap percentage (BP, right). Black dots at the tree nodes indicate PP = 1 and BP = 100%. Support values of PP < 0.95 and BP < 70% are omitted. Oblique transverse lines on the branches mark how many times the latter were shortened for the illustration. The newly obtained sequences are in bold. GenBank accession numbers for SSU, 5.8S and LSU rDNAs are separated by comma. Solid contigs of rDNA operon are marked with only one accession number. Sequences assembled from the available transcriptomic data are marked with SRA numbers or A if accession is not in SRA (see M&M). Absent sequences are marked with -.

The Bayesian (Fig. 4) and ML (Supplementary fig. S2) trees for the rDNA concatenate had identical topologies and showed the monophyly of major terrestrial and marine eugregarine groups: Actinocephaloidea, Gregarinoidea, Stenophoroidea, Stylocephaloidea, Ancoroidea, Cephaloidophoroidea, and Lecudinoidea, although some of them received low support. The gregarines from centipedes obtained in this study formed a fully supported (posterior probabilities, PP = 1 and bootstrap percentages, BP = 100) monophyletic group together with unidentified gregarines whose sequences were obtained from either environmental sequencing data or centipede transcriptomic data contaminated with gregarine parasites (see M&M). The dactylophorids (Dactylophoroidae) formed a compact and fully supported clade (PP = 1, BP = 100) sister to the Trichorhynchidae clade (PP = 1, BP = 100) that included *Trichorhynchus efeykini* and another gregarine from a scutigeromorph *Scutigerina weberi*. We designate this highly supported union of dactylophorids and trichorhynchids as a new superfamily—the Dactylophoroidea. In our phylogenies the Dactylophoroidea were sister to the clade uniting Actinocephaloidea and Stylocephaloidea with high to moderate support (PP = 1, BP = 85).

## Discussion

The number of gregarine species described from centipedes is less than a third of that from millipedes^2^. This disparity might be an indication of greater morphological similarity of gregarines from centipedes, which might mask their true diversity, but it might also be simply due to the fact that centipedes are more difficult to catch and identify than millipedes, leading to a bias in their representation. Thus far, *Trichorhynchus pulcher* was the only described member of the Trichorhynchidae, with several reported findings being attributed to the same species. This likely is an underestimate of the true diversity in the family, since the reported findings of* T*. *pulcher* demonstrate some morphological differences. Several authors describe and depict the gregarines with ovoid nuclei^13,22,23^. Hoshide^24^ does not describe the appearance of *Trichorhynchus* gregarines found in *Thereuonema clunifera* and *T*. *tuberculata* in Japan, but according to the Fig. 256 in the article, the author observed a *Trichorhynchus* sp. with apparently a round nucleus. The gregarines found in *Scutigera coleoptrata* in Slovenia also had apparently round nuclei (Fig. 1–3 in^25^). The gregarine described in the current study was assigned to the genus *Trichorhynchus* because it shares the key features with *Trichorhynchus pulcher*, such as a lobed epimerite anchored close to the basal lamina of the host epithelium, a well-developed neck—an elongated anterior end of protomerite deeply immersed is the host intestinal epithelium, cell surface covered with multiple cytopilia, and both parasites infect scutigeromorph centipedes. However, we classified *Trichorhynchus efeykini* sp. n. as a new species because its appearance differs from the previously described *T. pulcher*, including the shape of trophozoites and the protomerite length to total length ratios: 1:4 to 1:7 for *T*. *pulcher*^22^ versus 1:2.5 to 1:3 for *T*. *efeykini*. Although molecular data are still lacking to adequately assess the diversity of trichorhynchids, we can assume that gregarine parasites of scutigeromorphs may differ greatly, since the rDNA sequences assembled here for *T*. *efeykini* (5563 bp) and another putative *Trichorhynchus* species found in the transcriptomic data of a South African scutigeromorph centipede *Scutigerina weberi* (5588 bp) display only 90% identity across the whole operon. Therefore, more sequenced isolates from different locations and host species are needed to elucidate the true diversity and distribution of trichorhynchids.

In our phylogenies (Fig. 4, Supplementary fig. S2) the trichorhynchids formed a fully supported group that branched sister to the dactylophorids *E. hispida*, *G. gracilis* and related sequences. The trichorhynchid branch diverged closer to the root of the Trichorhynchidae + Dactylophoridae lineage, with both dactylophorids and trichorhynchids forming relatively tight clusters of more closely-related sequences. Thus, our molecular data support the separation of *Trichorhynchus* from the Dactylophoridae proposed by Ormières and colleagues on the basis of morphological characteristics^13^.

Nevertheless, the fully supported sister relationship between Trichorhynchidae and Dactylophoridae (Fig. 4), an evolutionary lineage of exclusive parasites of centipedes, deserves the taxonomical rank of superfamily, similarly to other major lineages of terrestrial gregarines^5,6^. We propose the name Dactylophoroidea after the first described family Dactylophoridae. The Dactylophoroidea have radiated through carnivorous hosts—centipedes, which may have conditioned the evolution of their well-developed attachment apparatus: protoepimerite^2^—a lobate epimerite and a well-developed protomerite neck deeply immersed in the host intestinal epithelium in trichorhynchids, and rhizoids in dactylophorids (Fig. 1, Supplementary fig. S1,^2,9,11,13^). We can cautiously assume that the syzygy in Dactylophoroidea should form late, since late frontal syzygies have been observed in some dactylophorids^9,26,27^ and no syzygies have been reported in trichorhynchids to date. The Dactylophoroidea have radiated through hosts most commonly found in moist environments. This is reflected in their method of the gametocyst dehiscence that occurs with the help of a pseudocyst, a cytoplasmic residuum left over from sporogony. The pseudocyst dilates when exposed to a humid atmosphere. In the dactylophorids the pseudocyst causes the rupture of the hyaline gametocyst wall, while in the trichorhynchids there is no hyaline wall and the pseudocyst opens the gametocyst along the dark equatorial ring^2,13,15^. Oocysts are reported cylindrical in the dactylophorid and trichorhynchid species studied in this respect^9,14,15,26–28^.

Ribosomal DNA phylogeny shows that gregarines from millipedes and centipedes form different lineages that are not sister to each other (Fig. 4). All the gregarine sequences found in millipede transcriptomes and the described gregarines from millipedes sequenced to date are placed in the Stenophoroidea clade that is sister to the Gregarinoidea^7,29^. The centipede-specific parasites—dactylophorids and trichorhynchids are more closely related to the Stylocephaloidea and Actinocephaloidea. The latter superfamily is also known to include gregarines from centipedes in addition to parasites of other invertebrates^10,24^. The gregarines sequenced by Yazaki and colleagues^30^ confirm this observation, since the scolopendromorhp *Scolopocryptops rubiginosus* parasite misidentified as *Stenophora* sp. (“gregarine ex *Scolopocryptops rubiginosus*” in our tree) was in fact a dactylophorid closely related to *Echinomera*, and the two gregarines from millipedes *Orthomorpha* sp. and *Parafontaria laminata armigera* both grouped within the Stenophoroidea (Fig. 4). The closer relationship between Dactylophoroidea and Actinocephaloidea is also confirmed by multiprotein phylogenies^30,31^ where the dactylophorid misidentified as *Stenophora* sp. grouped with actinocephaloids *Monocystis agilis*, *Ascogregarina taiwanensis* and a gregarine from *Teleopsis dalmanni* with a full support.

Based on all of the above, we propose to establish a new superfamily Dactylophoroidea that includes two families: Dactylophoridae and Trichorhynchidae.

Taxonomy

Phylum Apicomplexa Levine, 1970

Subphylum Sporozoa Leuckart, 1879

Class Gregarinomorpha Grassé, 1953

Order Eugregarinida Léger, 1900

Superfamily Dactylophoroidea superfam. nov.

Syzygy late frontal in dactylophorids^9,26,27^, unknown in trichorhynchids. Gametocyst dehiscence by pseudocyst ^2,13,15^. Oocysts cylindrical ^9,14,15,26–28^. In centipedes.

Family Dactylophoridae Léger, 1892

Family Trichorhynchidae Ormières, Marquès & Puisségur, 1977

*Trichorhynchus* Schneider, 1882

*Trichorhynchus efeykini* Miroliubova et Kudriavkina sp. n.

Trophozoites are narrowly obpyriform, 149.7 ± 9.1 μm long (min. 85 μm and max. 242.9 μm) and 48.8 ± 2.4 μm wide (min. 29.5 μm and max. 69 μm); the protomerite length to total length ratio is 1:2.5 to 1:3; deutomerite of the same width as protomerite near the septum; a lobed epimerite at the anterior end of an elongated protomerite neck; nucleus spherical, in any part of the deutomerite; nucleolus eccentrically placed; the nucleolus diameter to the nucleus diameter ratio is 1:2; cell surface with multiple cytopilia.

DNA SEQUENCE. GenBank PP657470.

TYPE LOCALITY. Hòn Giao mount, Khánh Hòa, Vietnam (12° 13′10″ N 108° 43′ 5″ E).

TYPE HABITAT. Terrestrial.

TYPE HOST. *Thereuopoda longicornis* Fabricius, 1793 (Chilopoda: Scutigeromorpha).

LOCATION IN HOST. Intestine.

TYPE (syntype) MATERIAL. A gold sputter-coated SEM stub with host gut pieces with attached and detached gregarines, 9 slides with histological sections stained with Ehrlich’s hematoxylin, a specimen of parasite cells and host material (intestine) fixed in 96% ethanol, and the host body (without viscera) fixed in 96% ethanol have been deposited in the collection of The Center for Parasitology IPEE RAS; Fig. 1 (this publication) shows some of the syntypes.

LSID: urn:lsid:zoobank.org:act:F975009C-2150-455D-B2AA-0DF15A850482.

ETYMOLOGY: named in honor of Boris Efeykin (junior researcher, IPEE RAS), who collected the infected individual of *Thereuopoda longicornis*.

## Materials and methods

Four individuals of *Scolopendra cingulata* Latreille, 1829 were collected in Koktebel (44°57′ 14.1″ N 35° 14′ 09.5″ E) in May 2021. Nineteen individuals of *Lithobius forficatus* Linnaeus, 1758 were collected from Timiryazevsky Park in Moscow (55° 48′ 29.7″ N 37° 33′ 04.4″ E) in May–July 2021. Five individuals of *L. forficatus* were collected near the Zvenigorod Biological Station of Moscow State University, Moscow region (55° 41′ 53.9″ N 36° 43′ 14.2″ E) in July 2021. One individual of *Thereuopoda longicornis* Fabricius, 1793 (Scutigeromorpha) was collected on the Hòn Giao mount, Khánh Hòa, Vietnam (12°13′ 10″ N 108°43′ 5″ E) in June 2023.

Centipedes were dissected in the insect-adapted Ringer’s solution (0.650% NaCl, 0.025% KCl, 0.025% CaCl2, 0.025% NaHCO3). Gregarine parasites were isolated from the host’s intestines with fine tip needles and plastic pipettes under MBS-1 and MBS-10 stereomicroscopes (LOMO, Russia). For electron microscopy, small fragments of the host intestine with attached gregarines and free gregarines were fixed with 1% glutaraldehyde (v/v) in 0.05 M cacodylate buffer, pH 7.35, final osmolarity 210 mOsm (4 °C, 2 h), then rinsed with the cacodylate buffer and post-fixed with 1% osmium tetroxide (w/v) in the cacodylate buffer (4 °C, 2 h). After fixation, the sample was dehydrated in an ethanol series (15%, 30%, 50%, 70%, 80%, 96%). For histological preparations, fragments of the host intestine with attached gregarines were fixed with Bouin’s solution, rinsed two times with distilled water and dehydrated in ethanol series (30%, 50%, 70%, 96%, 100%). Using paraffin-celloidin method 5 μm thick sections were made with the Leica RM-2265 microtome (Leica Microsystems, Germany) and stained with Ehrlich’s hematoxylin^32^.

For molecular phylogenetic studies, all isolated gregarines (forcedly detached trophozoites and gamonts) were washed thrice in fresh portions of the insect-adapted Ringer’s solution before fixation/lysis. Twenty-five individuals of each *Echinomera hispida* from Timiryazevsky Park (TP isolate), twenty-five individuals *Grebnickiella gracilis*, and one hundred individuals of *Trichorhynchus efeykini* sp. n. were fixed in 96% ethanol. Seven individuals of *E. hispida* from the Zvenigorod Biological Station (ZBS isolate) were lysed in 1X reaction buffer (SMART-Seq v4 Ultra Low Input RNA Kit, Japan). The samples were then used entirely for DNA or RNA extraction.

Live parasites were photographed with different cameras by means of bright-field microscopy: a DCM35 digital camera (SCOPETEK, PRC) connected to a MBI-3 light microscope (LOMO, Russia) for *G. gracilis*, a Carl Zeiss AxioCam b/w R1.1 412–311 digital camera connected to a Zeiss Axiostar plus light microscope (Carl Zeiss, Germany) for *E. hispida*, and an ADF STD16 digital camera connected to an ADF B30 light microscope (ADF, China) for *Trichorhynchus efeykini* sp. n. The following measurements were taken for *Trichorhynchus efeykini* sp. n.: TL—total length; PL—length of protomerite; DL—length of deutomerite; PWM—maximum width of protomerite; PWE—width of protomerite at equatorial axis; DWM—maximum width of deutomerite; DWE—width of deutomerite at equatorial axis^33^.

The gregarines fixed with glutaraldehyde and osmium tetroxide were transferred from 96% ethanol to a mixture of 96% ethanol and acetone (1:1, v/v), followed by three changes of pure acetone and displacement of acetone by liquid carbon dioxide, followed by critical point drying. After that, the samples were mounted on stubs, sputter-coated with gold/palladium, and examined under scanning electron microscopes: a JSM–6380LA (JEOL, Japan) for *G. gracilis* and *E. hispida,* and a Tescan MIRA3 LMU (TESCAN, Czech Republic) for *Trichorhynchus efeykini* sp. n.

For *E*. *hispida* TP isolate and *G*. *gracilis*, DNA was extracted with WLB + lysis buffer and proteinase K^34^ and further purified with the Diatom DMA Prep 100 kit (Galart-Diagnostikum, Russia). The rDNA fragments (18S rDNA, ITS1, 5,8S rDNA, ITS2, 28S rDNA) were amplified with Encyclo PCR kit (Eurogen, Russia) in a total volume of 20 μl in a thermal cycler Applied Biosystems MiniAmp (Thermo Scientific, USA).

18S rDNA were amplified using forward 5′-GTATCTGGTTGATCCTGCCAGT-3′ and reverse 5′-GGAAACCTTGTTACGACTTCTC-3′ primers. ITS2 regions were amplified using forward 5′-GTACACACCGCCCGTCGCTC-3′ and reverse 5′-GACTCCTTGGTCCGTGTTTCAAGACG-3′ primers. 28S rDNA was amplified using two pairs of primers: forward 5′-GACCCGCTGAAYTTAAGCATAT-3′ and reverse 5′-ACATTCAGAGCACTGGGCAG-3′, and forward 5′-GTCACTTCGGGAWAAGGATTGGCT-3′ and reverse 5′-TTCTGACTTAGAGGCGTTCAG-3′. PCR protocol: block lid temperature—100 °C, initial denaturation 95 °C—2 min 30 s, denaturation 95 °C—30 s, annealing 60–65 °C—30 s, elongation 72 °C—2 min 30 s; steps 2–4 were repeated 44 times, final extension 72 °C—10 min.

For *Trichorhynchus efeykini* sp. n., total RNA was extracted with RNAqueous-Micro Total RNA Isolation Kit (Invitrogen, USA). For *E. hispida* ZBS isolate and *Trichorhynchus efeykini* sp. n., the cDNA libraries were prepared using the SMART-Seq v4 Ultra Low Input RNA Kit (Takara Bio, Japan). 15.5 million paired-end 100 bp reads for *E. hispida* ZBS isolate were generated with Illumina HiSeq 4000. 79.7 million paired-end 150 bp reads for *Trichorhynchus efeykini* sp. n. were generated with Illumina NovaSeq 6000. The rDNA operon sequences (SSU rDNA, ITS1, 5.8S rDNA, ITS2, LSU rDNA) of *E. hispida* ZBS isolate and *Trichorhynchus efeykini* sp. n. were assembled from the transcriptomic data using NOVOPlasty assembler^35^. Ribotypes were identified in the rDNA assemblies by mapping library reads with Bowtie2^36^.

In order to improve the sampling of gregarines related to the known dactylophorids, we performed BLAST searches in the nr database (NCBI) and available metagenomic data (NCBI Sequence Read Archive, accessions: PRJEB25197) for environmental sequences related to these gregarines. Further, we searched for gregarine contamination in the NCBI Sequence Read Archive available transcriptomic data of different centipedes. The contamination was found and gregarine rDNA sequences were extracted from three libraries: *Cryptops anomalans* (SRX1747805), *Scolopendra cingulata* (SRX760100), and *Scutigerina weberi* (SRX1637773). In order to increase the number of terrestrial gregarine operational taxonomic units (OTUs) that contain 5.8S and LSU sequences, we extracted rDNA sequences from available gregarine transcriptomic data: SRX9590447, SRX9590448, SRX5759500, SRX554020, SRX5759502, SRX5759508, and https://doi.org/10.5061/dryad.6m905qfz6 for a gregarine from a centipede *Scolopocryptops rubiginosus*. In addition, we extracted gregarine rDNA sequences from available metagenomic data (SRX7069788) and transcriptomic data of two insect hosts (SRX7069788 and SRX1828758).

Three rDNA alignments (the SSU, 5.8S, and LSU) with different representations of apicomplexans (222, 107, and 102 OTUs respectively) were generated using the MAFFT online service employing the E-INS-i alignment method^37^. Poorly aligned regions and columns containing few nucleotides were removed from the alignments using a custom mask resulting in three alignments: 1,530 bp for the SSU, 145 bp for the 5.8S, and 2654 bp for the LSU rDNA. A total of 59 OTUs, including eugregarines, related environmental sequences, a cryptosporidian, and coccidians as an outgroup were selected for phylogenetic inference; their rDNAs were concatenated resulting in a 4,329 nucleotide site alignment.

Bayesian inference (BI) was performed with MrBayes 3.2.6^38^ utilizing the resources of the CIPRES web server^39^. The analysis was performed under the GTR + Г + I model with 8 rate categories for the SSU and LSU rDNA partitions, and with 4 rate categories for the 5.8S rDNA partition. The chain heating coefficient (temp) was set to 0.2. The inference was done with two independent runs of four MCMC, and the consensus tree was built with a 50% burn-in after 10 million generations, and the tree sampling frequency of 0.001. The average standard deviation of split frequencies at the end of computations was 0.002945. Maximum likelihood (ML) analysis was performed with the IQ-TREE 2.1.2^40^ using the GTR + I + G8 + F model for the SSU and LSU rDNA partitions and the GTR + I + G4 + F for the 5.8S rDNA partition. The computations were performed with 1000 nonparametric bootstrap^41^ replicates for estimation of branch support. The Bayesian and ML consensus trees had an identical topology; the ML support values were superimposed on the Bayesian tree for the illustration.

The predicted secondary structures of ITS2 sequences of *E. hispida* isolates were generated with the RNAfold web server (http://rna.tbi.univie.ac.at/) at the temperature setting of 37 °C under default parameters. To determine whether *E. hispida* isolates belong to different or the same species, compensatory base changes in the secondary structures of the ITS2 sequences were searched^16–19^.

## Supplementary Information

Supplementary Information.

## Data Availability

Sequence data that support the findings of this study have been deposited in the GenBank with the primary accession codes: PP657469 (*Echinomera hispida* ZBS isolate SSU rDNA, ITS1, 5.8S rDNA, ITS2, and LSU rDNA), PP654190 (*Grebnickiella gracilis* SSU rDNA, ITS1, 5.8S rDNA, ITS2, and LSU rDNA), PP657470 (*Trichorhynchus efeykini* sp. n. SSU rDNA, ITS1, 5.8S rDNA, ITS2, and LSU rDNA); PP659854 (*Echinomera hispida* TP isolate SSU rDNA); PP659855 (*Echinomera hispida* TP isolate ribotype 1 ITS2), PP659857 (Echinomera* hispida* TP isolate ribotype 2 ITS2), PP659856 (*Echinomera hispida* ZBS isolate ribotype 2 ITS2). The datasets generated and analyzed during the study are available from the corresponding author on request.
