# Supplementary material for: Parasites specific to centipedes form a new major lineage of terrestrial gregarines
Source: Sci Rep. 2025 Jan 2;15:192. doi: 10.1038/s41598-024-83990-4 (PMC11695991; doi:10.1038/s41598-024-83990-4)
Supplement: Supplementary file 1 — Supplementary Information. [file 41598_2024_83990_MOESM1_ESM.pdf]

Parasites specific to centipedes form a new major lineage of terrestrial gregarines

Tatiana S. Miroliubova[1,2*], Kirill V. Mikhailov[3,4], Timur G. Simdyanov[5], Vladimir V. Aleoshin[3,4,5], Đinh Thế Dũng[2], Aleksandra I. Kudriavkina[1,2]

[1]Severtsov Institute of Ecology and Evolution, Russian Academy of Sciences, Leninsky ave. 33, Moscow, 119071 Russian Federation; [2]Joint Vietnam-Russia Tropical Science and Technology Research Center, Hanoi, Vietnam; [3]Belozersky Institute for Physico-Chemical Biology, Lomonosov Moscow State University, Leninskiye Gory 1, bldg. 40, Moscow, 119991 Russian Federation; [4]Kharkevich Institute for Information Transmission Problems, Russian Academy of Sciences, Bolshoy Karetny ln. 19, bldg. 1, Moscow, 127051 Russian Federation; [5]Faculty of Biology, Lomonosov Moscow State University, Leninskiye Gory 1, bldg. 12, 119234, Moscow, Russian Federation. *email: provorosenok@gmail.com

Supplementary Figure S1. A-H — series of histological sections of *Thereuopoda longicornis* intestinal epithelium with a *T. efeykini* trophozoite attached. Abbreviations: d — deutomerite; ep — epimerite; n — nucleus; ne — neck; nu — nucleolus; p — protomerite; s — septum.

Supplementary Figure S2. Maximum-likelihood tree of eugregarines with an alignment of 59 concatenated SSU, 5.8S and LSU rDNA sequences (4,329 sites). Tree node support values: non-parametric bootstrap percentage. The newly obtained sequences are in bold. GenBank accession numbers for SSU, 58S and LSU rDNAs are separated by comma. Solid contigs of rDNA operon are marked with only one accession number. Sequences assembled from the available transcriptomic data are marked with SRA numbers or A if accession is not in SRA (see M&M). Absent sequences are marked with -.

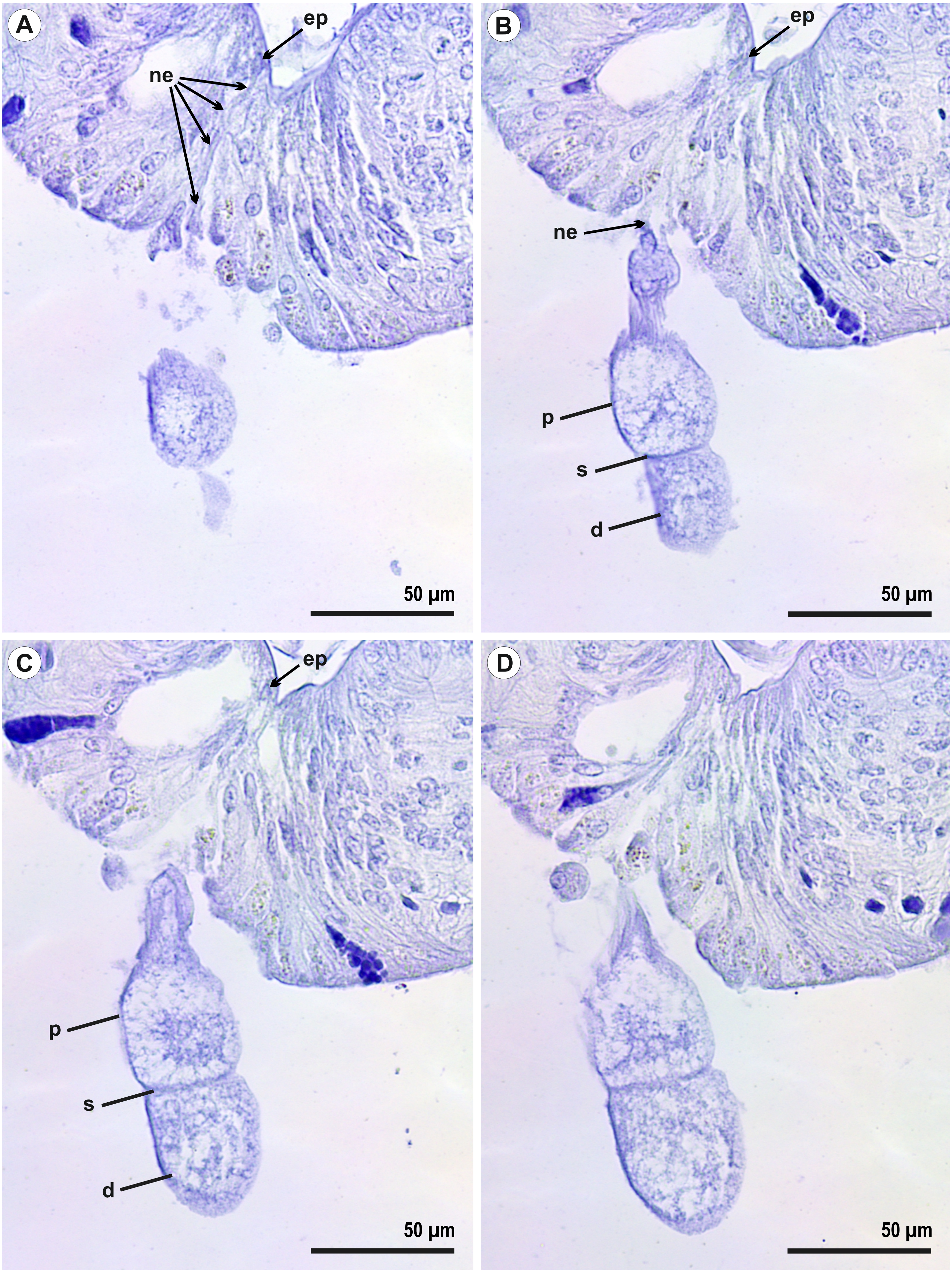

Supplementary Fig.S1 part 1

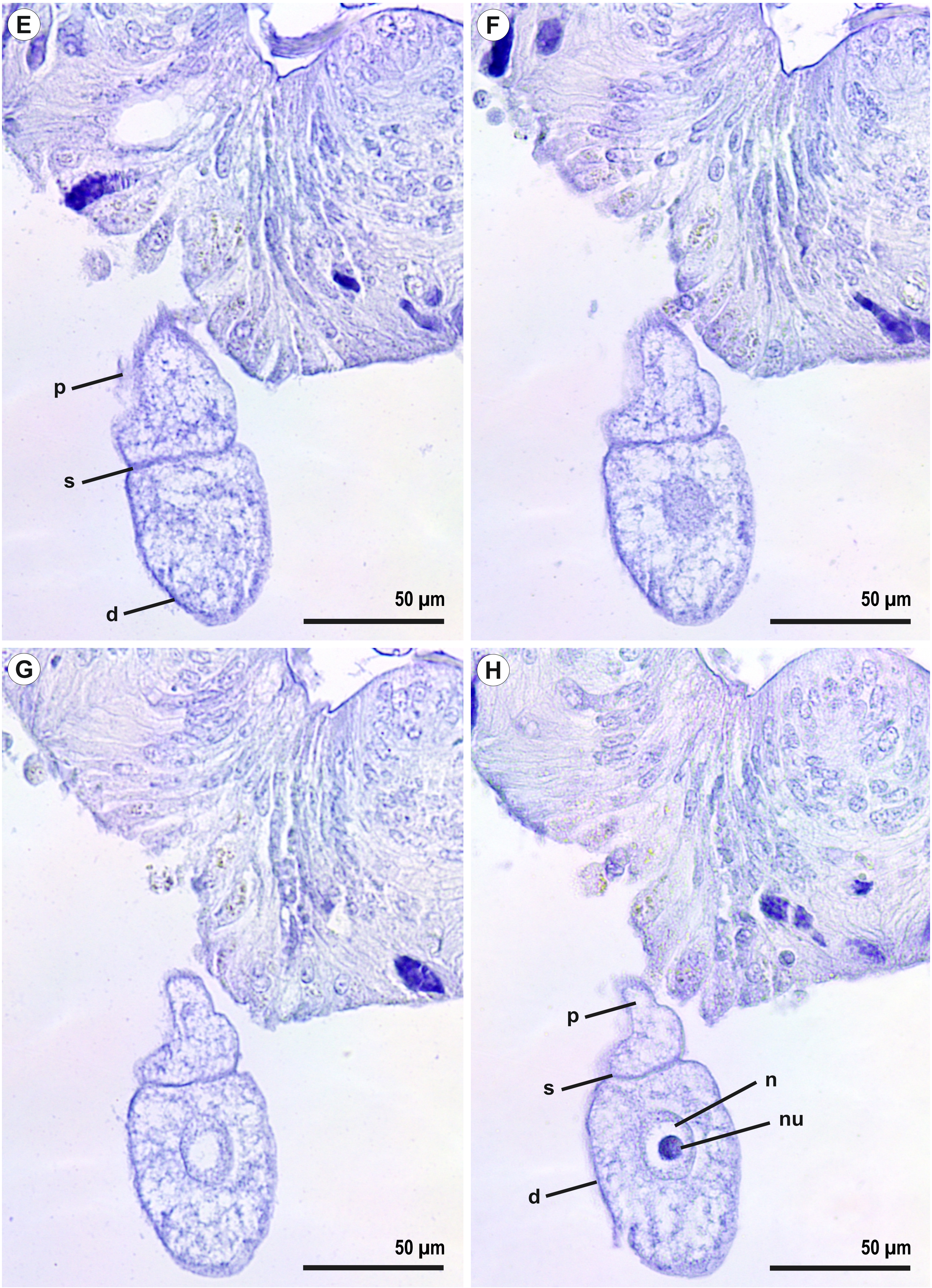

Supplementary Fig.S1 part 2

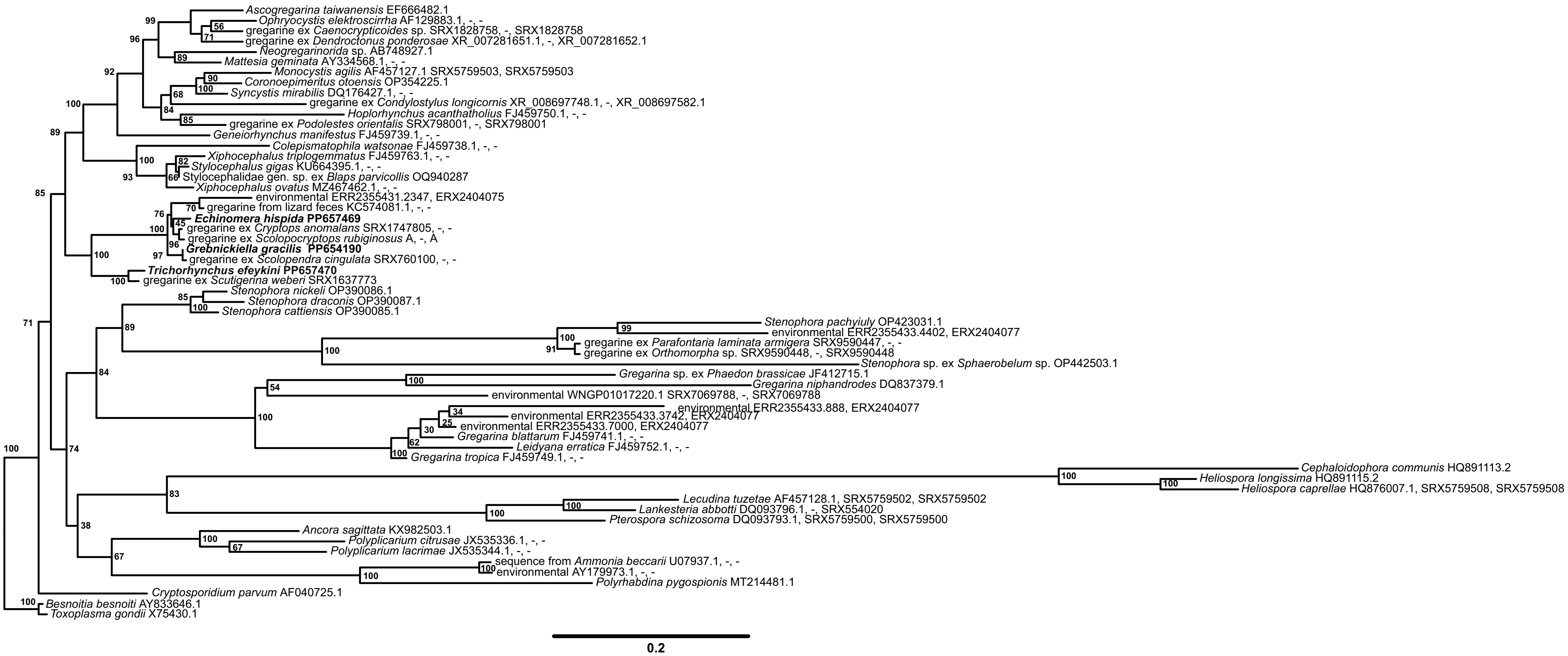

Supplementary Fig.S2
